# Applications of Copper-Catalyzed Click Chemistry in Activity-Based Protein Profiling

**DOI:** 10.3390/molecules19021378

**Published:** 2014-01-27

**Authors:** Julianne Martell, Eranthie Weerapana

**Affiliations:** Department of Chemistry, Boston College, Chestnut Hill, MA 02467, USA; E-Mail: marteljb@bc.edu

**Keywords:** click chemistry, activity-based protein profiling, bioorthogonal chemistry, azide-alkyne cycloaddition

## Abstract

Activity-based protein profiling (ABPP) is a chemical proteomic technique that enables the interrogation of protein activity directly within complex proteomes. Given the dominant role of posttranslational modifications in regulating protein function *in vivo*, ABPP provides a direct readout of activity that is not attained through traditional proteomic methods. ABPP relies on the design of covalent binding probes that either target a specific enzyme or a class of enzymes with related function. These covalent warheads are coupled to either fluorophores or biotin groups for visualization and enrichment of these active proteins. The advent of bioorthogonal chemistries, in particular, the copper (I)-catalyzed azide-alkyne cycloaddition (CuAAC), has benefitted the field of ABPP by achieving the following: (1) replacing bulky reporter groups with smaller alkyne or azide groups to promote cell permeability; (2) adding modularity to the system such that a single probe can be diversified with a variety of reporter groups without the need to develop new synthetic routes; and (3) enabling the conjugation of complex linkers to facilitate quantitative proteomic analyses. Here, we summarize recent examples of CuAAC in ABPP that serve to illustrate the contribution of bioorthogonal chemistry to advancing discoveries in this field.

## 1. Introduction

**Figure 1:**
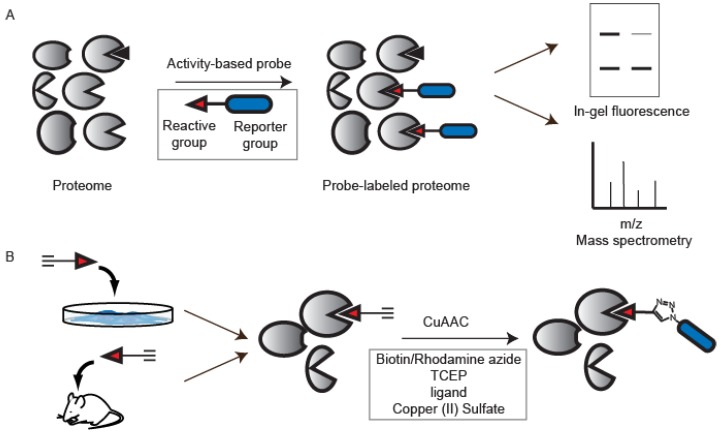
Activity-based protein profiling (ABPP). (**A**) Typical ABPP experiments utilize activity-based probes (ABPs) that comprise a reactive group bound to a reporter group (inset). ABPs label only active enzymes within a protein mixture, and protein labeling can be visualized by in-gel fluorescence and labeled proteins can be identified using mass spectrometry; (**B**) For CuAAC-mediated ABPP, the reporter group is replaced by a bioorthogonal ligation handle, typically an alkyne. Probe labeling can be performed *in vivo*, and the reporter tag conjugated *ex vivo* using CuAAC conditions.

The ability to directly monitor protein activity within a native biological system is essential to understanding the dysregulation of proteins in disease. Standard proteomic methods such as two-dimensional gel electrophoresis and mass spectrometry can effectively quantify protein abundance in complex proteomes [1]. However, due to the diverse and dynamic posttranslational modifications that regulate protein activity *in vivo* [2], these methods do not afford insight into the fraction of active protein within a given system. To address these limitations, the field of activity-based protein profiling (ABPP) was developed to complement existing genomic and proteomic technologies [3,4,5,6]. ABPP relies on the use of chemical probes that selectively react with the active site of a specific protein, or a class of proteins with shared functional properties [7]. These activity-based probes (ABPs) are typically composed of a reactive electrophile to covalently modify an active-site residue, and a reporter group, such as a fluorophore for visualization by in-gel fluorescence, or a biotin for both western blot analysis and avidin enrichment prior to mass-spectrometry (MS)-based identification (Figure 1A) [8,9]. 

**Figure 2:**
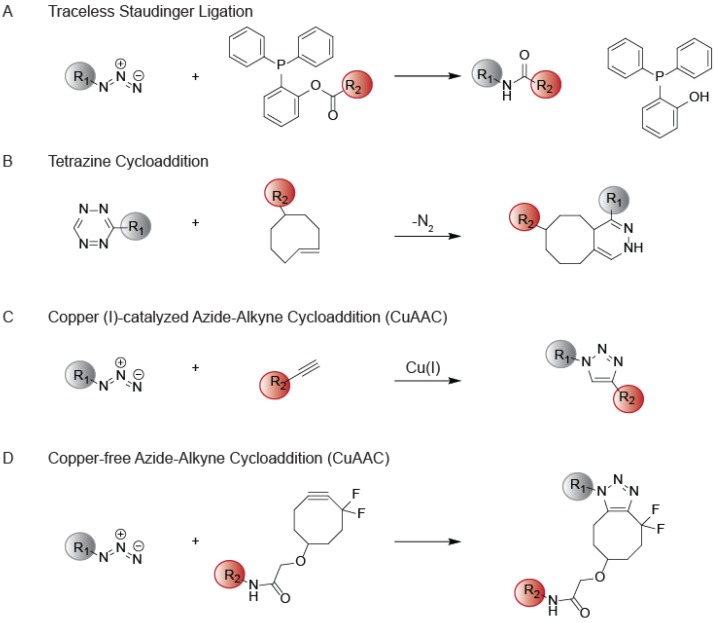
Bioorthogonal Reactions. (**A**) The traceless Staudinger Ligation couples azides with triarylphosphines to generate an amide linkage; (**B**) The Tetrazine Cycloaddition utilizes a 1,2,4,5-tetrazine and a strained diene; (**C**) The CuAAC reaction forms a 1,4-disubstituted 1,2,3-triazole from an azide-alkyne cycloaddition promoted by Cu(I); (**D**) The copper-free variant of the azide-alkyne cycloaddition utilizes a strained alkyne to accelerate the reaction.

While useful for *in vitro* analysis of protein activities, these bulky reporter groups can hinder cellular uptake and protein affinity when administered *in vivo*. To circumvent these issues, ABPs can be functionalized instead with sterically inconspicuous bio-orthogonal handles to facilitate attachment of reporter groups *ex vivo* (Figure 1B) [10,11,12,13]. An ideal bioorthogonal reaction involves the rapid and selective coupling of two biologically inert coupling partners under physiological conditions [14]. The Staudinger ligation was one of the first bioorthogonal reactions to be developed, and is based on the modified Staudinger reaction between azides and triarylphosphines (Figure 2A) [15,16]. More recently, the tetrazine ligation has found wide utility and couples a highly strained *trans*-cyclooctene to functionalized tetrazines (Figure 2B) [17,18,19]. The most widely used click chemistry reaction is the copper (I)-catalyzed azide-alkyne cycloaddition (CuAAC) between an azide and a terminal alkyne to generate a 1,4-disubstituted 1,2,3-triazole (Figure 2C) [20,21]. Concerns about the use of a cytotoxic copper species to catalyze the reaction promoted the development of a copper-free variant of this reaction that utilizes a strained alkyne to accelerate the reaction (Figure 2D) [22,23,24]. Furthermore, the development of ligands that simultaneously act to reduce reactive oxygen species produced during typical CuAAC reactions further ameliorate toxicity issues associated with this reaction and enable live-cell labeling [25]. All of these bioorthogonal reactions have been applied to the field of ABPP and many of these studies have been reviewed previously [13]. Here we will specifically focus on the application of the CuAAC bioorthogonal reaction to ABPP. CuAAC has advanced the field of ABPP by expanding the enzyme classes that can be targeted by ABPs, enabling cellular and *in vivo* studies and providing technological platforms to quantitatively monitor protein activities in complex biological systems. 

## 2. The Development of CuAAC and Early Applications to ABPP

The CuAAC reaction is a derivative of the Huisgen 1,3-dipolar cycloaddition [26] that fuses azides and alkynes to form triazoles (Figure 2C). This reaction is ideal for biological applications due to the high stability of azides to water, ambient oxygen and a wide-variety of synthetic transformations [27]. The synthetic ease of incorporating azides and alkynes into biological probes, coupled with the minimal steric disruption caused by these functionalities, has further promoted the use of CuAAC in biological applications. The initial Huisgen cycloaddition required high temperatures and formed a mixture of 1,4- and 1,5-triazole regioisomers, but addition of a copper(I) catalyst provides exclusively 1,4-disubstituted-1,2,3-triazoles at room temperature, at a wide range of pH values, and in high yield [20,27]. The biocompatibility of this reaction was originally demonstrated through decoration of viral capsids [21]. The CuAAC reaction was first utilized in the field of ABPP to couple an azide-derivatized phenyl sulfonate ester ABP (PS-N_3_) to an alkyne-bearing rhodamine moiety (Rh-≡) [10]. The PS-N_3_ probe labeled GSTO 1-1 proteins in cell lysates more efficiently than the standard rhodamine-tagged phenyl sulfonate probe (PS-Rh). Furthermore, PS-N_3_ was shown to facilitate *in vivo* ABPP, as cells and animals treated with PS-N_3_ showed robust protein labeling upon administration of the CuAAC reagents *ex vivo* [10]. Further optimization of this platform revealed that the use of rhodamine-azide (Rh-N_3_) greatly reduced the high background labeling of proteins that was observed with Rh-≡, although with lower kinetics of labeling [11]. This initial foray into “tag-free” ABPP clearly demonstrated the key advantages of this platform, which includes better distribution of the probe in cells and animals, improved access to protein active sites, and streamlined probe synthesis to create a single modular probe that can be linked to a variety of reporter groups. Since then, “tag-free” ABPP has been employed in a variety of studies that have resulted in the expansion of ABPP into new enzyme classes.

## 3. Alkyne-Tagged ABPs for the Serine Hydrolase Family

**Figure 3:**
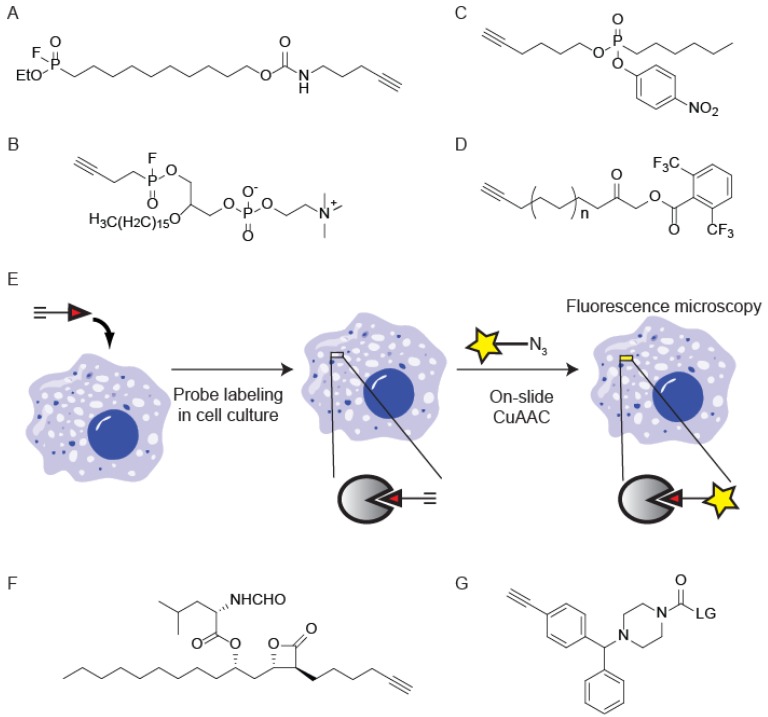
CuAAC-compatible ABPs for serine hydrolases. (**A**) Fluorophosphonate-alkyne (FP-≡) is a cell-permeable pan-serine hydrolase probe; (**B**) A phosphatidylcholine-based probe for profiling serine phospholipases; (**C**) A nitrophenyl-phosphonate (NP) ester-based probe for lipase profiling in cells; (**D**) An acyloxymethylketone (AOMK) probe for profiling lipid-associated proteins; (**E**) Workflow for imaging subcellular localization of active enzymes in cells using CuAAC-ABPP. Probe labeling is followed by cell fixation and addition of CuAAC reagents; (**F**) An Orlistat analog that labels fatty acid synthase; (**G**) The general scaffold for a library of carbamate-based probes for selective serine hydrolase labeling (LG = leaving group).

The first class of enzymes targeted for ABPP studies were the serine hydrolases (SHs), which comprise a large and diverse family of enzymes that perform numerous roles in physiological (e.g., blood coagulation, inflammation, angiogenesis) and pathological (e.g., emphysema, cancer) processes [28]. This family of enzymes is characterized by an active site serine residue that is rendered nucleophilic by the presence of a catalytic dyad or triad involving proximal Lys, Asp and His residues [29]. ABPs for this family of enzymes were derived from fluorophosphonates (FPs), which were known to be mechanism-based inhibitors that mimic the enzyme-substrate tetrahedral intermediate and covalently trap the active site serine [30,31]. Most ABPP studies for SHs are performed using biotin or rhodamine tagged FP [32], but CuAAC has shown utility in the profiling of these enzymes with the generation of a “tag-free” FP-alkyne (FP-≡) (Figure 3A) [33,34]. This probe was shown to label serine proteases with greater affinity than the reporter-tag functionalized FP probes [33]. More importantly, the vastly improved cell permeability of FP-≡ facilitates profiling of SH activity directly in living cells. This improved cell permeability enabled the development of a competitive ABPP platform to screen inhibitor selectivity directly within a cellular milieu instead of in lysates where information pertaining to subcellular localization is disrupted [33]. In addition to pan-serine hydrolase ABPs, FP-based probes for specific SHs have been developed with inclusion of an alkyne handle for CuAAC. These include phosphatidylcholine-based probes where the reactive FP-warhead is positioned at either *sn*-1 or *sn*-2 positions (Figure 3B). These probes were shown to label functional subclasses of serine phospholipases [34]. Unlike bulky biotin or rhodamine reporter groups, the alkyne causes minimal steric perturbation and better mimics the acyl chain in the endogenous substrates for these phospholipases. 

Electrophilic groups other than the fluorophosphonate can also be used to covalently modify the active-site serine of SHs. These other serine-reactive electrophiles include nitrophenyl-phosphonate esters (NP), acyloxymethylketones (AOMK) and carbamates. Many ABPs containing these electrophiles have been functionalized with azides or alkynes for “tag-free” ABPP using CuAAC. The NP and AOMK reactive groups have been incorporated into alkyne-functionalized fatty acid-based chemical probes for profiling lipid-associated proteins (Figure 3C,D) [35,36]. The minimal steric encumbrance of the alkyne facilitates cellular uptake and enables activity-based profiling of a variety of fatty acid-modifying proteins in living cells [35,36]. The NP fatty acid probes were additionally used to image the subcellular localization of active lipases. This was achieved by performing CuAAC to incorporate a fluorophore for protein visualization after fixation of the cells (Figure 3E) [36]. In a similar imaging study, an alkyne-functionalized analog of Orlistat, a β-lactone-based covalent inhibitor of fatty acid synthase (FASN), was used to image the localization of FASN during Hepatitis C Virus infection (Figure 3F) [37]. The SH-reactive carbamate reactive group was incorporated into a library of alkyne-functionalized small molecules (Figure 3G) and led to the discovery of a hexafluoroisopropyl carbamate that specifically labels two endocannabinoid hydrolases (monoacylglycerol lipase (MAGL) and α, β hydrolase-6 (ABHD6)) in cells [38]. 

In addition to the lipases, serine proteases have also been targeted by “tag-free” ABPP. Pan serine protease ABPs for in-cell labeling have been developed from the irreversible inhibitor 4-(2-aminoethyl)benzenesulfonyl fluoride (AEBSF) [39], iso-coumarins that target subclasses of serine proteases [40], peptidyl aldehydes and phosphonates that specifically label prolyl oligopeptidase [41,42], and β-lactones that inhibit rhomboid proteases [43]. 

## 4. CuAAC-Compatible Covalent Probes for Other Enzyme Families

**Figure 4:**
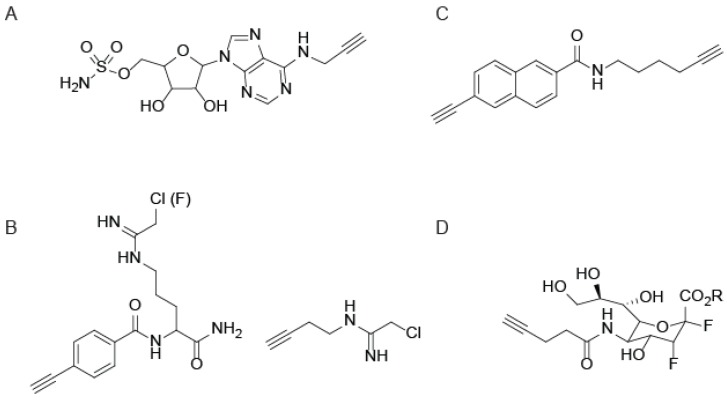
Activity-based probes for diverse enzyme families. (**A**) An ABP for the ubiquitin-conjugating enzyme, UBE1; (**B**) Chloro(and fluoro) amidine-based probes for protein arginine deiminases (PADs) and dimethylarginine dimethylaminohydrolase (DDAH); (**C**) 2EN-ABP, a probe for cytochrome P450 enzymes; (**D**) A sialic acid derivative for covalent labeling and imaging of sialidases.

Although the serine hydrolases were the subject of some of the earliest ABPP studies, “tag-free” ABPs are constantly being developed for diverse enzyme families. As with the SH probes, the use of CuAAC in each of these cases improves protein reactivity and cell permeability. One family of enzymes recently targeted by CuAAC-mediated ABPP facilitates studies into the ubiquitin and ubiquitin-like protein (UBL) signaling pathways [44]. A series of ubiquitin-conjugating enzymes tag proteins with ubiquitin for targeted degradation by the proteasome [45], including UBL activating enzymes (UBE1), UBL conjugating enzymes (UBE2), and UBL ligases (UBE3). Since UBE1 enzymes dictate the activity of the entire ubiquitin-signaling pathway, a click-chemistry compatible ABP for UBE1 enzymes was developed to measure UBE1 activity inside cells [44]. The UBE1 ABP (termed ABP1; Figure 4A) was designed to mimic and bind the AMP binding site of the UBE1-UBL thioester complex, positioning a nucleophilic sulfamate group for attack of the UBE1-UBL thioester to form a covalent adduct. This cell permeable ABP1 efficiently labels active UBE1 enzymes in numerous cancer cell lines, allowing for profiling the selectivity of UBE1 inhibitors within cells, and facilitating the discovery of previously unknown UBL proteins [44].

The epoxide-containing natural product E-64 has been shown to potently inhibit several papain-like cysteine proteases by alkylating the active site cysteine [46,47]. A set of E-64 derivatives functionalized with azide (N_3_Le) or alkyne (≡Le) handles were generated for *in vivo* labeling of plant tissues [48]. In contrast to biotin-tagged E-64 [47], N_3_Le and ≡Le efficiently labeled proteases in living plant tissues [48]. In this same study, a similar approach was utilized to label the proteasome in plants. Several ABPs for the proteasome have been developed for bioorthogonal reactions outside of CuAAC [13,49,50,51] One of those proteasome ABPs was adapted for CuAAC by incorporation of an azide functional group for CuAAC-mediated coupling to biotin after treatment of living plant tissues. These studies enabled the screening of inhibitors for selectivity in plant tissues [48]. 

Outside of the cysteine proteases and ubiquitinating enzymes, several other enzymes with nucleophilic active-site cysteines have been targeted for CuAAC-mediated ABPP. One such family is the protein arginine deiminases (PADs), which catalyze the conversion of arginine residues to citrulline in the presence of calcium [52]. Several members of the PAD family have been shown to play critical roles in cell signaling pathways, including apoptosis and cell differentiation, as well as transcriptional regulation by modifying arginine residues in the tails of histones [52]. PAD probes were constructed around mechanism-based inactivators that contain a haloacetamidine (Cl or F) to covalently modify the active site cysteine [53]. Alkyne-functionalized PAD ABPs (Figure 4B) were shown to be more selective and efficient at labeling PAD4 in cells compared to the biotin and fluorescein-tagged counterparts [54]. This increased efficiency of labeling is attributed to the rapid degradation of PAD4 in cell lysates, which is circumvented by in-cell labeling with CuAAC-compatible probes. A similar alkyne-functionalized chloroacetamidine probe (Figure 4B) was shown to label the related enzyme dimethylarginine dimethylaminohydrolase (DDAH) [55]. Similar to the PADs, DDAH activity is diminished in lysates due to inactivation by a variety of effector molecules, therefore *in vivo* labeling using CuAAC-compatible probes serves to preserve enzyme activity [55]. 

Cytochrome P450 enzymes are central to the metabolism of numerous drugs, xenobiotics, and endogenous metabolites, and understanding the subset of active P450 enzymes is essential to the drug development process [56]. A set of ABPs based on three different mechanism-based inhibitors, containing aryl alkynes (Figure 4C), propynyl groups, or furanocoumarin, was synthesized and demonstrated complementarity in profiling the entire family of human P450s [57]. This panel of P450 APBs enabled investigation into the effects of clinically used aromatase inhibitors on P450 activity [57]. The sterically inconspicuous alkyne was critical to these studies, as biotin or fluorophore-tagged probes are less similar to typical drug-molecules that are metabolized by the P450s and therefore are not substrates for these enzymes.

Lastly, CuAAC-mediated ABPP has been extended to profile members of the glycosidase family. The exoglycosidase neuraminidase (NA; also known as sialidase) is produced by many pathogens to facilitate invasion and immunological escape [58]. A 3-fluorosialyl fluoride (DFSA) probe (Figure 4D) was designed to specifically label NA as well as an ester-protected version (PDFSA) to enhance cell permeability and label NA *in vivo* [59]. Imaging of active NA in influenza virus-infected cells was accomplished by CuAAC-mediated conjugation of biotin-azide followed by staining with DyLight488-streptavidin. The NA inhibitor Oseltamivir (OS) competitively inhibits DFSA binding, yet OS-resistant strains such as H1N1 are able to bind DSFA even in the presence of OS, suggesting the application of the DFSA probe for detection of OS-resistant influenza strains [59].

## 5. Photo-crosslinking ABPs that Utilize CuAAC

**Figure 5:**
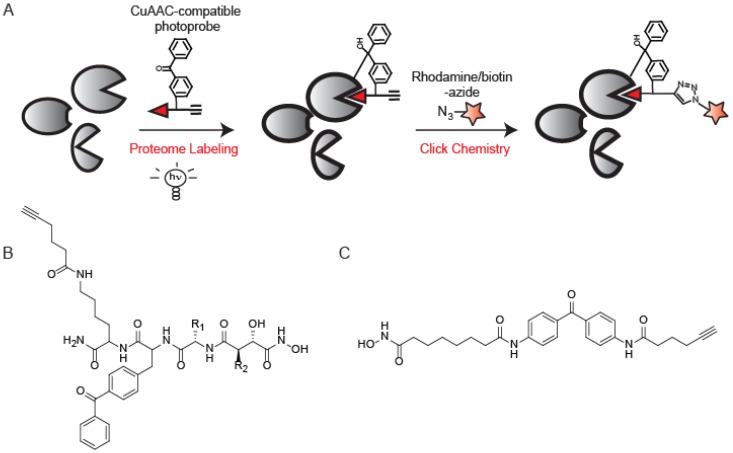
Photo-crosslinking CuAAC-compatible ABPs. (**A**) Standard workflow for a photo-crosslinking ABP involves probe labeling and UV irradiation followed by CuAAC to incorporate reporter groups; (**B**) A photo-crosslinking probe for metalloproteases; (**C**) A photo-crosslinking probe for histone deacetylases based on the known non-covalent inhibitor SAHA.

The design of ABPs typically relies on exploiting the mechanism of enzyme-substrate covalent interactions to irreversibly modify conserved active site nucleophiles [7]. However, many enzyme classes, including kinases, aspartyl proteases, and metalloproteases, do not contain an active site nucleophile that can be exploited for ABP design. To target these enzyme families, non-covalent small molecules can be functionalized with photo-crosslinking groups to facilitate covalent binding (Figure 5A). 

This strategy has proved to be highly successful in the design of probes for several different enzyme families. Metalloproteases (MPs) utilize a zinc-activated water molecule for catalysis, and it has been shown that small molecules containing hydroxamic acids (Hx) chelate to the catalytic zinc and bind with high affinity to MP active sites [60]. MP ABPs comprised of a Hx moiety to chelate to the active site zinc, a benzophenone for covalent modification, a variable binding domain to render specificity across the MP family, and an alkyne to conjugate reporter elements using CuAAC (Figure 5B) were evaluated [61]. Most MPs showed marked preference for labeling by alkyne-tagged probes over their rhodamine-tagged counterparts, with some showing affinity for only the alkyne versions [61]. Four probes were determined to target the majority of enzymes labeled by the library as a whole, creating an optimal “cocktail” of ABPs to profile enzyme activity across the MP family. 

A similar strategy was utilized to generate ABPs for the histone deacetylase (HDAC) family. HDACs are known to play a role in cancer but the study of HDAC activity is complicated by the presence of endogenous activating protein complexes that are disrupted upon cell lysis [62]. To develop an *in vivo* compatible ABP for HDACs, a known reversible inhibitor of HDACs, SAHA, was converted into an activity-based probe (SAHA-BPyne; Figure 5C) by installment of a benzophenone group and alkyne handle [63,64]. SAHA-BPyne showed enhanced sensitivity and selectivity when applied to living cells over cell lysates due to the myriad of endogenous factors that are likely absent or misplaced upon cell homogenization [63,64]. 

A photo-crosslinking ABP for Abelson tyrosine (Abl) kinase was developed from the core structure of Imatinib, an Abl kinase-specific drug for the treatment of Chronic Myelogenous Leukemia (CML) [65]. Incorporation of a benzophenone and a reporter element converted the non-covalent Imatinib into a covalent ABP. The rhodamine-tagged derivative was shown to be 200-fold less potent than the corresponding alkyne variant. This is attributed to poor accommodation of the bulky rhodamine within the kinase active site. Furthermore, non-specific labeling was enhanced in the rhodamine-tagged version [65], alluding to the benefits of CuAAC-mediated ABPP in this study.

Photo-crosslinking ABPs have also been applied to profile non-enzymatic activities such as that of the nicotinic acetylcholine receptor (nAChR), a neurotransmitter-gated ion channel [66]. An acetylcholine-mimic, benzophenone-alkyne-triethylammonium (BPyneTEA), was designed to create a probe that could differentiate between the closed, but activatable, and desensitized states. The ability to attach any reporter group to BPyneTEA via the alkyne handle allows for visualization of nAChR desensitization in live cells and characterization of protein interactions and PTMs associated with desensitization of nAChR during nicotine addiction and other disorders [66].

Outside of direct ABPP applications, photo-crosslinking CuAAC probes have been utilized to assist in target identification in drug discovery. One such example is the intermembrane aspartyl protease γ-secretase complex that cleaves amyloid precursor protein (APP) into neurotoxic Aβ42 peptides and is therefore a critical target in Alzheimer’s therapeutics [67]. γ-secretase modulators (GSMs) are attractive therapeutic agents as they reduce the formation of Aβ42 peptides without blocking the processing of other γ-secretase substrates, as observed with γ-secretase inhibitors (GSIs) [68]. Photo-reactive versions of GSMs and GSIs containing benzophenones and alkyne CuAAC handles were developed to identify targets of GSMs and better understand their mechanism of action [69,70,71]. Labeling by the different GSMs showed that multiple binding sites exist in the γ-secretase complex, each of which plays a role in regulating the activity and substrate binding of the complex. 

## 6. Applications of CuAAC in Mass Spectrometry-Based ABPP

**Figure 6:**
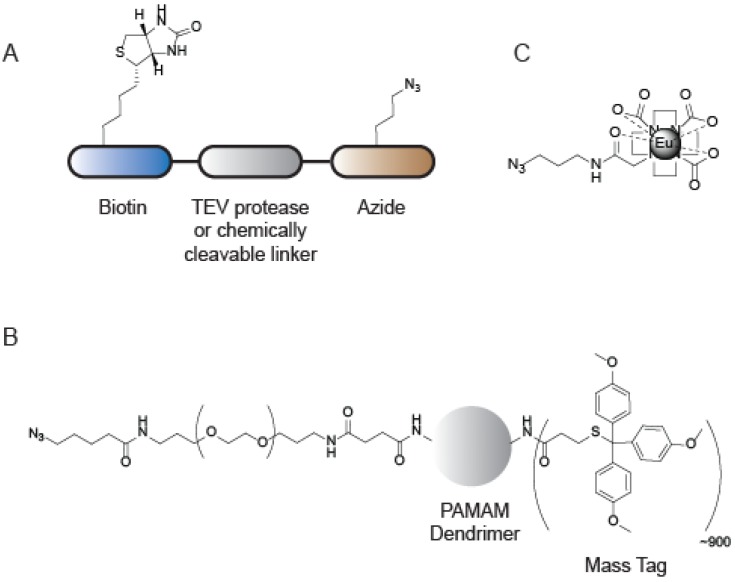
CuAAC-compatible tags for mass-spectrometry applications. (**A**) TEV-protease cleavable and chemically cleavable tags for identifying the site of labeling of ABPs; (**B**) Laser-cleavable mass tag for imaging mass spectrometry to identify the localization of active enzymes in cells and tissues; (**C**) Eu-labeled tag for ICP-MS based absolute quantification of active enzymes from a mixture.

SDS-PAGE and in-gel fluorescence only reveal a fraction of proteins targeted by APBs, and therefore, many mass spectrometry (MS)-based methods have emerged as gel-free alternatives that provide higher resolution and greater sensitivity [72]. One of the advantages of CuAAC-based ABPP methods is the ability to conjugate probe-labeled proteins to a variety of linkers that enable a diverse array of mass-spectrometry analyses. Typical MS-ABPP strategies utilize CuAAC to couple a biotin-azide for subsequent enrichment and identification of labeled proteins by mass spectrometry [12]. More advanced MS experiments involve the installment of a chemically or proteolytically cleavable linker that further allows for identification of the site of probe labeling, as well as the relative quantification of labeled proteins from two or more biological samples. A platform titled tandem orthogonal proteolysis (TOP)-ABPP utilizes CuAAC to append a linker comprised of a biotin group and an azide, separated by the recognition sequence for the tobacco-etch virus (TEV) protease (Figure 6A) [73,74]. 

Upon enrichment of probe-labeled proteins, on-bead trypsin digestion releases all but the probe-labeled peptides, which are selectively released from the beads by treatment with the TEV protease. MS analysis of this peptide mixture identifies the exact site of probe labeling from a complex proteome. This platform has been further modified by incorporating an isotopic label into the TEV linker, affording isotopically light and heavy variants that could be utilized to measure the extent of probe labeling across two different proteomes [75]. A further modification included the replacement of the protease-cleavable linker with a chemically cleavable azobenzene, allowing for faster cleavage of labeled peptides from the beads [76]. These platforms have expanded the scope and utility of ABPP through applications evaluating the amino-acid specificity of electrophiles used for ABPP [77], ranking cysteine residues in the proteome by their nucleophilicity so as to globally identify functional cysteines [75], and identifying residues subject to post-translational modification through reactive lipid species [78] and metal binding [79].

The utility of CuAAC in ABPP is further illustrated in a study that utilizes imaging mass spectrometry to study the distribution of active serine hydrolases in tissue [80]. In this study, tissue sections were treated with FP-≡, followed by incorporation of a mass-tagged dendrimer using CuAAC. The mass tags are connected to the dendrimer via laser-cleavable linkers (Figure 6B), thereby releasing the mass tags upon laser irradiation for direct analysis by MALDI-MS [80]. CuAAC has also been applied to quantification of serine protease activity using ICP/MS [81]. In this study, alkyne functionalized AEBSF analogs were modified with a europium-loaded azido-monoamide-DOTA using CuAAC (Figure 6C). Serine proteases that reacted with this probe were analyzed by ICP/MS allowing for absolute quantification of active serine proteases in a mixture [81]. 

CuAAC methods have greatly simplified these applications by circumventing the need to synthesize every ABP with the complex cleavable linkers pre-attached. The advent of CuAAC has resulted in a vastly modular process, where a single ABP can be evaluated through a variety of techniques by appending different tags using CuAAC. 

## 7. Conclusions

ABPP allows for selective labeling, visualization, and enrichment of active enzymes in a complex proteome. Initial ABPP probes were comprised of a reactive group directly conjugated to a reporter element such as a fluorophore or biotin. These bulky reporter groups precluded the application of ABPP to enzymes with binding sites that were unable to accommodate the reporter, and furthermore limited the use of ABP labeling in live cells and organisms due to the reduced cell permeability of the probe. Upon the advent of bioorthogonal chemistry, several mild and bio-compatible reactions were generated that enabled conjugation of the reporter element *ex vivo* after the initial probe-labeling was performed *in vivo*. Of these various bioorthogonal reactions, we focused specifically on CuAAC and its application to ABPP. CuAAC has significantly modularized ABPP approaches, whereby a single ABP can now be conjugated to a variety of reporter elements depending on the application of interest. Furthermore, CuAAC compatible probes are much smaller than reporter-tagged probes, facilitating probe labeling within the unperturbed environment of a live cell or animal. As emphasized in this review, many protein activities are modulated by protein complexes and other *in vivo* factors that are disrupted by homogenization, thereby CuAAC has greatly increased the number of enzyme families accessible to this technique. Furthermore, the small size of these clickable probes causes minimal steric interaction with enzyme active sites, thereby improving binding affinity and enhancing labeling. Lastly, the advent of CuAAC has facilitated the expansion of ABPP by coupling to advanced analytical platforms such as MALDI-imaging of active enzyme in tissue slices, absolute quantitation of active enzyme by ICP-MS, and direct identification and quantitation of the exact sites of probe labeling. Further advances in the efficiency of CuAAC, and the development of diverse clickable linkers to unite ABPP with a wide array of analytical techniques, will serve to expand the biological insight gained through this chemical proteomic technique.
